# istar: A Web Platform for Large-Scale Protein-Ligand Docking

**DOI:** 10.1371/journal.pone.0085678

**Published:** 2014-01-24

**Authors:** Hongjian Li, Kwong-Sak Leung, Pedro J. Ballester, Man-Hon Wong

**Affiliations:** 1 Department of Computer Science and Engineering, Chinese University of Hong Kong, Shatin, New Territories, Hong Kong; 2 European Bioinformatics Institute, Cambridge, United Kingdom; University of Erlangen-Nuremberg, Germany

## Abstract

Protein-ligand docking is a key computational method in the design of starting points for the drug discovery process. We are motivated by the desire to automate large-scale docking using our popular docking engine idock and thus have developed a publicly-accessible web platform called istar. Without tedious software installation, users can submit jobs using our website. Our istar website supports 1) filtering ligands by desired molecular properties and previewing the number of ligands to dock, 2) monitoring job progress in real time, and 3) visualizing ligand conformations and outputting free energy and ligand efficiency predicted by idock, binding affinity predicted by RF-Score, putative hydrogen bonds, and supplier information for easy purchase, three useful features commonly lacked on other online docking platforms like DOCK Blaster or iScreen. We have collected 17,224,424 ligands from the All Clean subset of the ZINC database, and revamped our docking engine idock to version 2.0, further improving docking speed and accuracy, and integrating RF-Score as an alternative rescoring function. To compare idock 2.0 with the state-of-the-art AutoDock Vina 1.1.2, we have carried out a rescoring benchmark and a redocking benchmark on the 2,897 and 343 protein-ligand complexes of PDBbind v2012 refined set and CSAR NRC HiQ Set 24Sept2010 respectively, and an execution time benchmark on 12 diverse proteins and 3,000 ligands of different molecular weight. Results show that, under various scenarios, idock achieves comparable success rates while outperforming AutoDock Vina in terms of docking speed by at least 8.69 times and at most 37.51 times. When evaluated on the PDBbind v2012 core set, our istar platform combining with RF-Score manages to reproduce Pearson's correlation coefficient and Spearman's correlation coefficient of as high as 0.855 and 0.859 respectively between the experimental binding affinity and the predicted binding affinity of the docked conformation. istar is freely available at http://istar.cse.cuhk.edu.hk/idock.

## Introduction

Protein-ligand docking predicts the preferred conformation and binding affinity of a small ligand as non-covalently bound to the specific binding site of a protein. Docking can therefore be used not only to determine whether a ligand binds, but also to understand how it binds. The latter is subsequently important to improve the potency and selectivity of binding. To date, there are hundreds of docking programs [1], [2]. The AutoDock series [3]–[5] is the most cited docking software in the research community, with over 5,000 citations according to Google Scholar. AutoDock has contributed to the discovery of several drugs, including the first clinically approved HIV integrase inhibitor [6]. Following its initial release, several parallel implementations were developed using either multithreading or computer cluster [7]–[9].

In 2009, AutoDock Vina [5] was released. As the successor of AutoDock 4 [4], AutoDock Vina significantly improves the average accuracy of the binding mode predictions while running two orders of magnitude faster with multithreading [5]. It was compared to AutoDock 4 on selecting active compounds against HIV protease, and was recommended for docking large molecules [10]. Its functionality of semi-flexible protein docking by enabling flexibility of side-chain residues was evaluated on VEGFR-2 [11]. To further facilitate the usage of AutoDock Vina, auxiliary tools were subsequently developed, including a PyMOL [12] plugin for program settings and visualization [13], a bootable operating system for computer clusters [14], a console application for virtual screening on Windows [15], and a GUI for virtual screening on Windows [16].

In 2011, inspired by AutoDock Vina, we developed idock 1.0 [17], a multithreaded virtual screening tool for flexible ligand docking. idock introduces plenty of innovations, such as caching receptor and grid maps in memory to permit efficient large-scale docking, revised numerical model for much faster energy approximation, and capability of automatic detection of inactive torsions for dimensionality reduction. When benchmarked on docking 10,928 drug-like ligands against HIV reverse transcriptase, idock 1.0 achieved a speedup of 3.3 in terms of CPU time and a speedup of 7.5 in terms of elapsed time on average compared to AutoDock Vina, making idock one of the fastest docking software.

Having released idock, we kept receiving docking requests from our colleagues and collaborators. They are mostly biochemists and pharmacologists, outsourcing the docking research to us after discovering pharmaceutical protein targets for certain diseases of therapeutic interest. Consequently, we had to grab the protein structure, do format conversion, define search space, set up docking parameters, and keep running idock in batch for months. Tedious enough, all the above work was done manually, resulting in very low research productivity. In order to automate large-scale protein-ligand docking using our idock, we have therefore developed a web platform called istar.

A few online docking platforms already exist. DOCK Blaster [18] investigates the feasibility of full automation of protein-ligand docking. It utilizes DOCK [19] as the docking engine and ZINC [20], [21] as the ligand database. It also utilizes PocketPickker (CLIPPERS) [22] for binding pocket identification. iScreen [23] is a compacted web server for TCM (Traditional Chinese Medicine) docking and followed by customized *de novo* drug design. It utilizes PLANTS [24]–[26] as the docking engine and TCM@Taiwan [27] as the ligand database. It also utilizes LEA3D [28] for *de novo* ligand design. FORECASTER [29] is a web interface consisting of a set of tools for the virtual screening of small molecules binding to biomacromolecules (proteins, receptors, and nucleic acids). It utilizes the flexible-target docking program FITTED [30] as docking engine. Nevertheless, the above platforms neither support fine-grained ligand selection based on molecular properties, nor be able to monitor job progress in real time. They also lack straightforward output of compound suppliers, a hurdle preventing users from purchasing high-rank compounds for further wet-lab verification. We aim to address these obstacles on our istar platform. Moreover, we strongly emphasize docking efficiency, which we believe is the most crucial factor for public large-scale docking platforms, so we try every endeavor to optimize our docking engine idock. Furthermore, we adopt the robust RF-Score [31] as a rescoring function for accurate prediction of binding affinity.

## Methods

In the following four subsections, we introduce our fast docking engine idock, our accurate rescoring function RF-Score, our modern web platform istar, and our experimental settings.

### Docking Engine idock

The input to idock includes a rigid receptor, a set of flexible ligands, and a cubic box, which is used to restrict the conformational space to a particular binding site of the receptor. The output from idock includes predicted conformations and their predicted binding affinity.

idock consists of two core components, a scoring function to predict binding affinity, and an optimization algorithm to explore the conformational space. idock inherits the same scoring function from AutoDock Vina. The idock score is made up of a conformation-dependent part and a conformation-independent part. The conformation-dependent part is a weighted sum of five terms over all the pairs of atoms 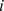 and 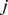 that can move relative to each other, excluding 1–4 interactions, i.e. atoms separated by three consecutive covalent bonds. The sum is calculated from equations (1) and (2) where 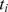 and 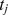 are the atom types of 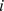 and 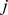 respectively, and 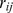 is their interatomic distance with a cutoff at 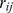 = 8Å. The five terms are calculated from equations (3) to (7) where 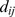 is the surface distance calculated from equation (8) where 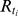 and 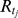 are the Van der Waals radii of 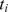 and 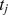 respectively. All the units are in Å. The first three terms account for steric interactions, the fourth term accounts for hydrophobic effect, and the fifth term accounts for hydrogen bonding. Metal ions are treated as hydrogen bond donors. The weighting coefficients are derived from linear regression on the PDBbind [32], [33] v2007 refined set (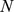 = 1,300). The optimization algorithm attempts to find the global minimum of 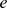 and other low-scoring conformations, which it then ranks.
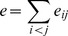
(1)

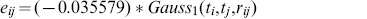


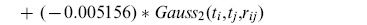


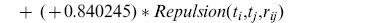


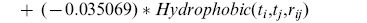


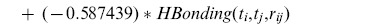
(2)

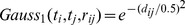
(3)

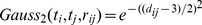
(4)

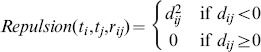
(5)

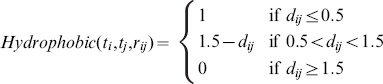
(6)

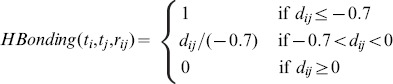
(7)

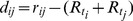
(8)


The conformation-dependent part can be seen as the sum of inter-molecular and intra-molecular contributions. Hence equation (1) can be rewritten into equation (9) where 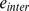 is the summation over all the heavy atom pairs between receptor and ligand, and 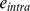 is the summation over all the non 1–4 heavy atom pairs of ligand.
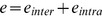
(9)


The conformation-independent part penalizes 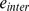 for ligand flexibility. The predicted free energy of the 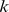
th conformation for output, denoted as 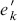, is calculated from equation (10) where 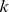 is the subscript for conformation, 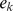 is the conformation-dependent score of the 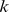
th conformation calculated from equation (1), 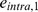 is the 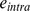 of the first, i.e. lowest-scoring conformation, 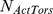 is the number of active torsions and 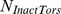 is the number of inactive torsions of the ligand. Note that 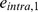, rather than 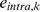, is subtracted in order to preserve the ranking.
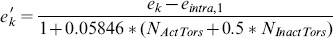
(10)


On one hand, in order to fast evaluate 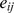, idock precalculates all its possible values. Note that 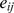 is essentially a function of three variables, namely 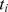, 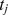, and 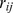, which have known lower and upper bounds. There are 15 heavy atom types implemented in idock, the pair of 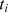 and 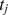 can thus have 120 ( = 15*16/2) different combinations. Since 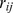 is cut off at 8Å, idock uniformly samples 16,384 points in the range [0, 8] and precalculates their 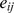 from equation (2). Subsequently, given a combination of 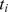, 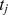 and 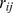, idock approximates the true value of 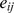 by table lookup rather than linear interpolation as used in AutoDock Vina.

On the other hand, in order to fast evaluate 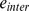, idock precalculates all its possible values by building grid maps. A grid map of atom type *t* is constructed by placing virtual probe atoms of atom type *t* along the X, Y, Z dimensions of the search box at a certain granularity. The 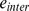 value of these probe atoms are precalculated from equation (2). Subsequently, given a sampled conformation, idock approximates the true values of 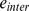 of ligand heavy atoms by table lookup rather than linear interpolation as used in AutoDock Vina. In fact, when we profiled AutoDock Vina, its linear interpolation of the 8 nearest corner probe atoms turned out to be a performance bottleneck because it involves 8 readings, 12 subtractions, 24 multiplications, and 7 additions. The grid granularity is hard-coded to be a coarse value of 0.375Å in AutoDock Vina, while in idock it is exposed as a program option for users to adjust accordingly and has a default fine value of 0.15625Å.

Likewise in AutoDock Vina, idock also uses Broyden-Fletcher-Goldfarb-Shanno (BFGS) [34] Quasi-Newton method for local optimization. In each BFGS iteration, a conformational mutation and a line search are taken, with each sampled conformation being accepted according to the Metropolis criterion. The number of iterations correlates to the complexity of the ligand regarding number of heavy atoms and number of torsions. BFGS approximates the inverse Hessian matrix, i.e. it uses not only the value of the scoring function but also its gradient, which are the derivatives of the scoring function with respect to the position, orientation and torsions of the ligand. Although both programs share similar optimization algorithms, their internal implementations differ. In idock, the BFGS local optimization stops if and only if no appropriate step length can be obtained by line search, thus increasing the probability of finding optimal local minimums. More optimization runs with fewer number of BFGS iterations are executed, better balancing high conformational diversity and short execution time.

idock introduces a novel feature that can automatically detect and deactivate certain torsions which are activated in the input file but indeed have no impact on the overall scoring, such as hydroxyl group – OH, amine group – NH_2_ or methyl group – CH_3_, because they only rotate the hydrogens and thus have no contributions to the idock score. idock is capable of re-classifying them as inactive torsions during parsing, thus reducing the dimension of variables to optimize in subsequent BFGS iterations.

idock encapsulates many more improvements. Please refer to its change log for a complete list of new features and bugfixes.

### Scoring Function RF-Score

RF-Score [31] is a member of a new class of scoring functions that use non-parametric machine learning approach to predict binding affinity in an entirely data-driven manner. RF-Score has been rigorously shown [31], [35] to perform better than 16 classical scoring functions in ranking protein-ligand complexes according to predicted binding affinity. It has also been shown to be useful in the discovery of new molecular scaffolds in antibacterial hit identification [36].

RF-Score is the first application of Random Forests [37] to predicting protein-ligand binding affinity. In RF-Score, each feature comprises the number of occurrences of a particular protein-ligand atom type pair interacting within a certain distance range. Four common atom types for the protein (i.e. C,N,O,S) and nine common atom types for the ligand (i.e. C,N,O,F,P,S,Cl,Br,I) constitute a vector of 36 features, and the distance cutoff is chosen to be as sufficiently large as 12Å to implicitly capture solvation effects.

The original version of RF-Score [31] is trained on PDBbind v2007 refined set less the core set (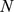 = 1,105). It grows each binary tree using the CART algorithm [38] without pruning from a bootstrap sample of the training data. It selects the best split at each node of the tree from a typically small number of randomly chosen features, and stops splitting a node with no more than 5 samples. The prediction from an individual tree is the arithmetic mean of its samples in the traversed leaf node. The performance of RF-Score does not vary significantly with the number of trees beyond a certain threshold, so we subscribe to the common practice of using 500 as a sufficiently large number of trees. The final prediction is the arithmetic mean of the individual predictions of all the trees in the forest.

We have re-trained the RF-Score on PDBbind v2012 refined set (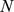 = 2,897) for prospective prediction purpose, and integrated it into our istar platform as an alternative option to re-score predicted conformations. We have also implemented a consensus score as the average effect of idock score and RF-Score. Mathematically speaking, equations (11) to (13) relate equilibrium constant 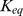 and dissociation constant 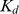 with Gibbs free energy 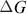, where 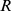 is gas constant (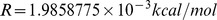) and 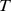 is absolute temperature.
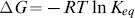
(11)

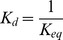
(12)

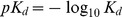
(13)


Assuming 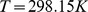 at room temperature, plugging equations (12) and (13) into (11) yields
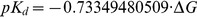
(14)


Equation (14) transforms the predicted free energy output by idock in 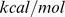 into binding affinity in 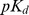 unit. The consensus score is thus defined in equation (15) so that it directly reflects the predicted potency in 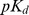 unit.
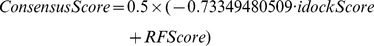
(15)


### Web Platform istar

Figure S1 shows the overall architecture of istar. On our istar website, the first section displays summary of existing jobs and the second section allows new job submission. A job comprises compulsory fields and optional fields. Compulsory fields include a receptor in PDB format, a search space defined by a cubic box, a brief description about the job, and an email to receive completion notification. Optional fields include nine ligand filtering conditions. The nine ligand filtering conditions are molecular weight, partition coefficient xlogP, apolar desolvation, polar desolvation, number of hydrogen bond donors, number of hydrogen bond acceptors, topological polar surface area tPSA, net charge, and number of rotatable bonds. These nine molecular descriptors are directly retrieved from our data source, i.e. the ZINC database [20], [21], in which the nine descriptors are already precalculated. Note that although molecular mass in Dalton unit may be a more appropriate descriptor than molecular weight in g/mol unit, we stick to the latter in order to maintain consistency with ZINC, in which the g/mol unit is used for molecular weight.

We have collected 17,224,424 ligands at pH 7 in mol2 format from versions 2012–04–26 and 2013–01–10 of the All Clean subset the ZINC database [20], [21] with explicit permission of its major developer and maintainer. The All Clean subset is constituted by applying strict filtering rules (http://blaster.docking.org/filtering), e.g. aldehydes and thiols have been removed. We have then converted the entire 17 million ligands in batch into PDBQT format as used by idock and the AutoDock series. The huge number of 17 million ligands should be sufficient in most cases. In case users need to screen their own ligand libraries, at present we recommend them run idock locally on their computers. We may consider allowing users to upload customized ligand libraries under certain constraints in future releases of istar.

istar supports ligand selection by desired molecular properties in a fine-grained manner and previewing the number of ligands to dock in real time (Figure S2). Users can move the nine sliders to filter ligands in the form of closed intervals. Only the ligands satisfying all the nine filtering conditions will be docked. Because of the relationship of logical and, in order to nullify a specific filtering condition, one may expand its closed interval to cover the entire possible range. We have set up default values of the lower and upper bounds of the nine molecular properties for novices to get started easily.

istar supports monitoring job progress in real time (Figure S3). We have composed a timer to automatically fetch and report the latest job progress every second without page refresh. Users can thus have a rough estimation in advance of how long the jobs will take and when the jobs will complete. This feature is particularly handy when the jobs are long running, which is usually the case of large-scale docking.

istar outputs verbose information in PDBQT format (Figure S4). The first REMARK line describes the ZINC ID, molecular weight (g/mol), partition coefficient xlogP, apolar desolvation (kcal/mol), polar desolvation (kcal/mol), number of hydrogen bond donors, number of hydrogen bond acceptors, topological polar surface area tPSA (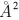), net charge, and number of rotatable bonds of a selected ligand. The second REMARK line describes the SMILES representation. The third REMARK line describes the number of suppliers followed by their names, which conform to the nomenclature as used by ZINC. The subsequent REMARK lines describe the free energy and ligand efficiency predicted by idock, putative hydrogen bonds, binding affinity predicted by RF-Score, and consensus score in 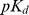 or 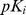 unit. Columns 71 to 76 of the ATOM lines describe the predicted free energy of each atom. The individual atom contribution to the overall score facilitates the detection of protein-ligand interaction hotspots, and thus assists in *de novo* ligand design.

At the moment, we have deployed a machine with Intel Xeon W3520 @ 2.66 GHz and 8GB DDR3 SDRAM to run the web server, and two identical virtual machines with Intel Xeon E5620 @ 2.40 GHz and 8GB DDR3 SDRAM to run the idock daemons, resulting in a docking speed of about one second per ligand. We have mounted a 2TB hard disk into our network file system to store docking jobs and results.

### Experimental Settings

We evaluated and compared idock ×86_64 v2.0 and AutoDock Vina ×86 v1.1.2 from the perspectives of rescoring, redocking and execution time on three datasets, which are PDBbind [32], [33], CSAR [39], [40] and ZINC [20], [21].

#### Datasets

The PDBbind v2012 dataset contains a diverse collection of experimentally determined structures carefully selected from PDB (Protein Data Bank) [41], [42]. For each complex, the experimental binding affinity (either dissociation constant 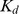, inhibition constant 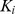, or half maximal inhibitory concentration 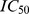) is manually collected from its primary literature reference, thus resulting in the general set of 9,308 complexes, with 7,121 being protein-ligand complexes. Out of them, the complexes with a resolution of 2.5Å or better, with known 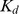 or 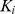 values, and with ligand containing merely the common heavy atoms (i.e. C, N, O, F, P, S, Cl, Br, I) are filtered to constitute the refined set of 2,897 complexes. These complexes are then clustered by protein sequence similarity using BLAST at a cutoff of 90%, and for each of the 67 resulting clusters with at least five complexes, the three complexes with the highest, median and lowest binding affinity are selected to constitute the core set of 201 complexes, whose experimental binding affinity spans 10 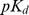 or 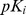 units.

The CSAR (Community Structure Activity Resource) NRC HiQ Set 24Sept2010 contains 343 diverse protein-ligand complexes selected from existing PDB [41], [42] entries which have binding affinity (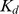 or 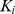) in Binding MOAD [43], [44], augmented with entries from PDBbind [32], [33]. Their binding affinity spans 12 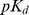 units.

The ZINC database contains over 21 million purchasable small molecules in popular MOL2 and SDF formats.

#### Benchmarks

In the rescoring benchmark, we evaluated the capability of RF-Score, AutoDock Vina and idock of predicting the binding affinity as close to the experimental binding affinity as possible given a crystal protein-ligand complex. We compared their rescoring performance to 18 other scoring functions on the PDBbind v2007 core set (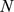 = 195). The test set was then extended to two larger datasets, i.e. the PDBbind v2012 [32], [33] refined set (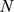 = 2,897) and the CSAR NRC HiQ Set 24Sept2010 (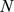 = 343) [39], [40] to enable a more comprehensive comparison.

In the redocking benchmark, we evaluated the capability of AutoDock Vina and idock of docking a randomized ligand conformation back to its crystal conformation as close as possible. We used the PDBbind v2012 [32], [33] refined set (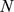 = 2,897), the PDBbind v2011 refined set (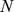 = 2,455), and the CSAR NRC HiQ Set 24Sept2010 (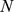 = 343) [39], [40], because they are the latest versions and contain the largest number of high-quality and diverse protein-ligand structures. We wrote a script to automatically define the search box first by finding the smallest cubic box that covers the entire ligand and then by extending the box by 10Å in all the three dimensions. Note that the 2rio entry of PDBbind contains two strontium ions, which are supported by idock but not by AutoDock Vina, we manually removed them before invoking AutoDock Vina. Both programs were also evaluated on the PDBbind v2012 core set (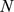 = 201) in order to find potential impact factors on their performance. We used root mean square deviation 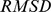 to measure the closeness between two conformations. The lower the 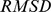 is, the closer the two conformations are. Usually the 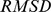 value is calculated between the crystal conformation and the docked conformation. Very often the 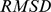 of 2.0Å is regarded as the positive control for correct bound structure prediction.

In the execution time benchmark, we collected 12 diverse proteins from the PDB (Protein Data Bank) database [41], [42], and 1000 ligands with a molecular weight of 200–300 g/mol, 1000 ligands with a molecular weight of 300–400 g/mol, and 1000 ligands with a molecular weight of 400–500 g/mol from the All Clean subset of the ZINC database [20], [21]. The 3,000 ligands were docked against the 12 proteins by AutoDock Vina and idock. Since AutoDock Vina can dock only one ligand in a run, three bash scripts containing 1,000 lines were executed instead, with each line being an execution of AutoDock Vina to dock one single ligand. The GNU Time utility v1.7 was used as a profiler.

The three benchmarks were carried out on desktop computers with Intel Core i5–2400 CPU @ 3.10GHz and 4GB DDR3 RAM under Mac OS X 10.7.4 Build 11E53. Arguments to AutoDock Vina and idock were left as default. By default, both programs output at most 9 predicted conformations per ligand.

## Results

### Rescoring Benchmark

Table 1 compares 21 scoring functions on the PDBbind v2007 core set (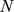 = 195). RF-Score [31], ID-Score [45], SVR-Score [46] and X-Score [47] are the only scoring functions whose training set do not overlap with the PDBbind v2007 core set. In terms of both Pearson's correlation coefficient and standard deviation, RF-Score performed the best, while AutoDock Vina and idock ranked 7th and 8th respectively, already outperforming the majority of commercial scoring functions.

**Table 1:** Comparison of 21 scoring functions on PDBbind v2007 core set (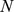 = 195).

Scoring function	*R_p_*	*R_s_*	SD
RF-Score	0.774	0.762	1.59
ID-Score	0.753	0.779	1.63
SVR-Score	0.726	0.739	1.70
X-Score::HMScore	0.644	0.705	1.83
DrugScoreCSD	0.569	0.627	1.96
SYBYL::ChemScore	0.555	0.585	1.98
AutoDock Vina	0.554	0.608	1.98
idock	0.546	0.612	1.99
DS::PLP1	0.545	0.588	2.00
GOLD::ASP	0.534	0.577	2.02
SYBYL::G-Score	0.492	0.536	2.08
DS::LUDI3	0.487	0.478	2.09
DS::LigScore2	0.464	0.507	2.12
GlideScore-XP	0.457	0.435	2.14
DS::PMF	0.445	0.448	2.14
GOLD::ChemScore	0.441	0.452	2.15
SYBYL::D-Score	0.392	0.447	2.19
DS::Jain	0.316	0.346	2.24
GOLD::GoldScore	0.295	0.322	2.29
SYBYL::PMF-Score	0.268	0.273	2.29
SYBYL::F-Score	0.216	0.243	2.35

Pearson's correlation coefficient 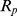, Spearman's correlation coefficient 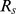 and standard deviation 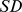 of the difference between predicted and experimental binding affinity on PDBbind v2007 core set (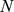 = 195). The scoring functions are sorted in the descending order of 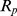. RF-Score, AutoDock Vina and idock rank 1st, 7th and 8th respectively in terms of Pearson's correlation coefficient 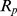. RF-Score, ID-Score, SVR-Score and X-Score are the only scoring functions whose training set do not overlap with the PDBbind v2007 core set. The statistics for AutoDock Vina and idock are reported in this study and the statistics for the other 19 scoring functions are collected from [31], [45], [46], [48].

**Figure 1:**
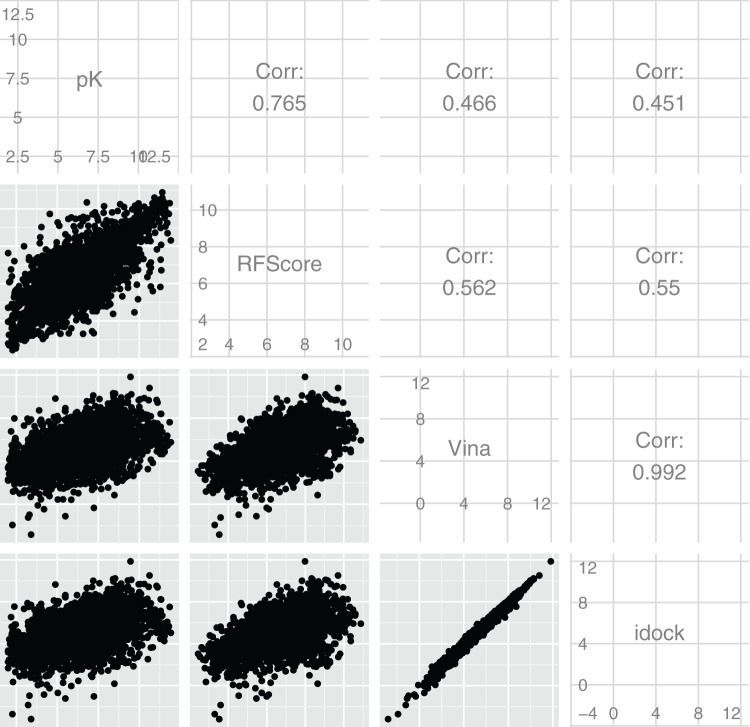
Pairwise correlations of experimental binding affinity and predicted binding affinity by RF-Score, AutoDock Vina and idock on the PDBbind v2012 refined set (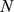 = 2,897). Values are in 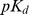 or 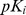 unit. The three scoring functions are all trained on the PDBbind v2007 refined set (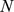 = 1,300). 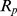 = 0.765, 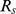 = 0.755, 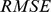  = 1.26, 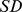  = .26 for RF-Score, 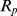 = 0.466, 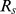 = 0.464, 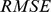  = 1.74, 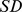  = 1.74 for Vina, and 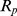 = 0.451, 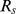 = 0.453, 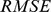  = 1.75, 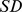  = .75 for idock.

**Figure 2:**
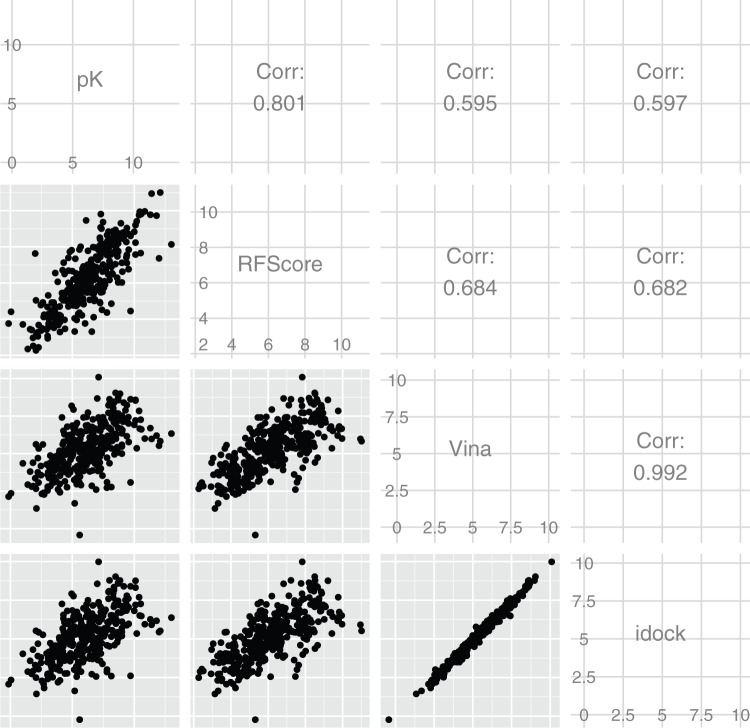
Pairwise correlations of experimental binding affinity and predicted binding affinity by RF-Score, AutoDock Vina and idock on the CSAR NRC HiQ Set 24Sept2010 (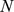 = 343). Values are in 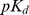 or 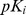 unit. The three scoring functions are all trained on the PDBbind v2007 refined set (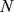 = 1,300). 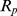 = 0.801, 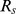 = 0.795, 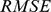  = 1.34, 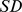  = 1.34 for RF-Score, 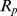 = 0.595, 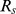 = 0.612, 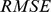  = 1.79, 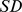  = 1.79 for Vina, and 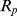 = 0.597, 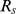 = 0.613, 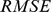  = 1.79, 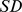  = 1.79 for idock.

Figure 1 plots the pairwise correlations amongst experimental binding affinity and predicted binding affinity by RF-Score, AutoDock Vina and idock on the PDBbind v2012 [32], [33] refined set (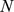 = 2,897) as it is the latest version. Since both AutoDock Vina and idock are trained on the PDBbind v2007 refined set (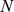 = 1,300), in order to make a fair comparison, in this benchmark we have re-trained RF-Score on the same training set. On one hand, the re-trained RF-Score managed to predict the binding affinity accurately with Pearson's correlation coefficient 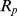 = 0.765, Spearman's correlation coefficient 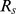 = 0.755, root mean square error 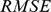  = 1.26, and standard deviation 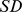  = 1.26. On the other hand, although AutoDock Vina and idock claimed to do well in conformation prediction, they could not predict binding affinity very accurately (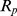 = 0.466, 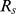 = 0.464, 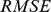  = 1.74, 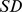  = 1.74 for AutoDock Vina, and 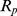 = 0.451, 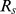 = 0.453, 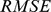  = 1.75, 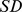  = 1.75 for idock), a very common obstacle in the entire computational chemistry community. As expected, the correlation between binding affinity predicted by AutoDock Vina and idock is very close to 1 because of their identical scoring function but different numerical approximation methods [17]. As can be seen from Figure 2, the above observations also apply to the results on the CSAR NRC HiQ Set 24Sept2010 (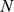 = 343) [39], [40].

### Redocking Benchmark

Figures S5, S6, S7 and S8 visualize the redocking results of four protein-ligand complexes selected from the PDBbind v2012 refined set. Table 2 shows the success rates of idock and AutoDock Vina under various conditions regarding the 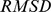 values between the crystal and docked conformations. Given a redocking case, 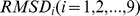 refers to the 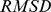 value between the crystal conformation and the 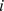
th docked conformation, i.e. the one with the 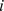
th highest predicted binding affinity, while 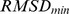 refers to the 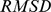 value between the crystal conformation and the closest docked conformation, i.e. the one with the minimum 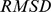 value. 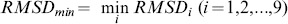. The condition 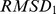  =  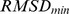 therefore tests for how many percent the docked conformation with the highest predicted binding affinity actually turns out to be the closest one among the 9 predicted conformations. It can be seen that the success rates of idock are comparable to, albeit slightly lower than, AutoDock Vina, and the success rates on the CSAR NRC HiQ Set 24Sept2010 are consistently higher than the PDBbind v2012 and v2011 refined sets, probably because the scoring function performs well on carefully refined structures. Using a RMSD value of 2.0Å, a publicly accepted positive control for correct bound structure prediction, both programs managed to predict a conformation sufficiently close to that of the co-crystallized ligand as the first conformation in over half of the cases, without any manual tweaking of the protein model.

**Table 2:** Redocking success rates.

	PDBbind v2012		PDBbind v2011		CSAR NRC HiQ	
Condition	idock	Vina	idock	Vina	idock	Vina
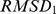 = 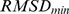	49%	53%	47%	54%	59%	71%
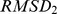 = 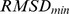	15%	16%	16%	14%	18%	13%
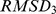 = 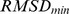	8%	7%	8%	8%	4%	4%
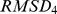 = 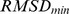	6%	6%	6%	5%	7%	3%
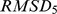 = 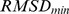	5%	4%	5%	5%	3%	1%
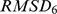 = 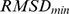	5%	3%	5%	4%	3%	3%
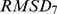 = 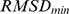	4%	4%	5%	4%	2%	2%
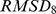 = 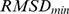	5%	3%	4%	3%	3%	2%
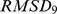 = 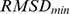	4%	3%	4%	3%	1%	2%
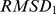 <0.5 Å	10%	12%	11%	12%	20%	21%
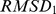 <1.0 Å	26%	31%	29%	31%	45%	47%
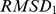 <1.5 Å	43%	47%	45%	47%	61%	67%
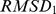 <2.0 Å	56%	60%	57%	59%	71%	75%
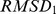 <2.5 Å	61%	65%	62%	65%	75%	79%
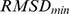 <0.5 Å	12%	15%	14%	15%	27%	26%
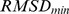 <1.0 Å	35%	40%	39%	40%	60%	55%
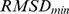 <1.5 Å	61%	65%	64%	65%	82%	84%
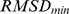 <2.0 Å	72%	79%	74%	78%	88%	92%
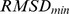 <2.5 Å	77%	85%	80%	84%	91%	94%

Redocking success rates of idock and AuoDock Vina on the PDBbind v2012 refined set (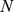 = 2,897), the PDBbind v2011 refined set (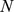 = 2,455), and the CSAR NRC HiQ Set 24Sept2010 (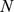 = 343) under various conditions regarding the 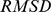 (Root Mean Square Deviation) values between the crystal and docked conformations. By default, both programs output 9 predicted conformations per ligand. 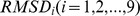 refers to the 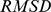 value between the crystal conformation and the 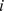
th docked conformation, i.e. the one with the 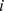
th highest predicted binding affinity, while 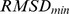 refers to the 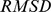 value between the crystal conformation and the closest docked conformation, i.e. the one with the minimum 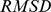 value. 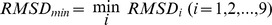. In conclusion, idock has a slightly higher conformation generation error than AutoDock Vina.

**Figure 3:**
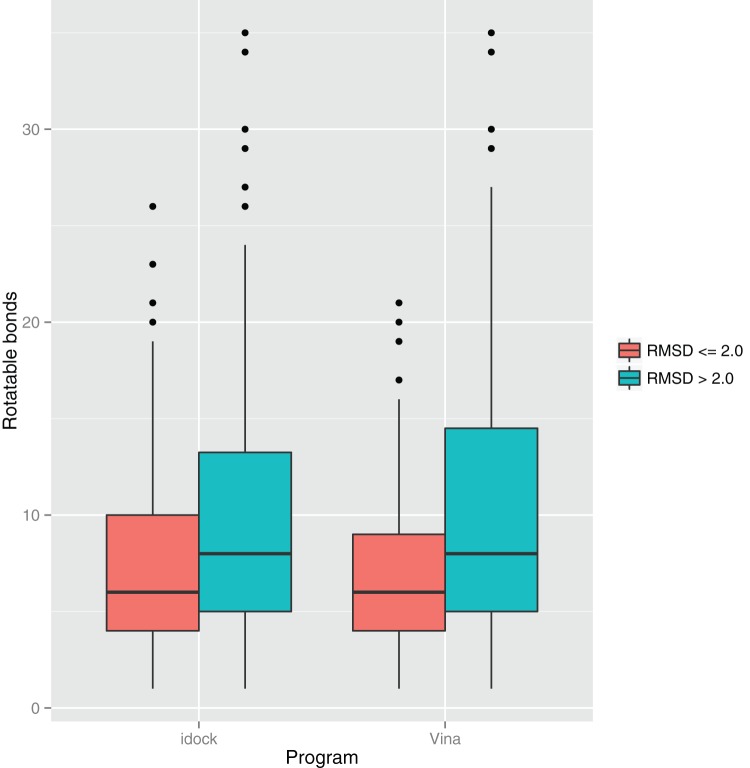
Impact of number of rotatable bonds of the ligand on the success rates of idock and AutoDock Vina benchmarked on PDBbind v2012 core set (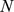 = 201). Very often the 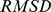 of 2.0Å is regarded as the positive control for correct bound structure prediction. Out the 201 cases, there are 109 and 114 successful cases for idock and AutoDock Vina respectively. The average number of rotatable bonds of the ligand in successful cases are 7.52 and 7.30 respectively for idock and AutoDock Vina. The average number of rotatable bonds of the ligand in unsuccessful cases are 10.36 and 10.82 respectively for idock and AutoDock Vina. Docking a ligand with no greater than 10 rotatable bonds has a higher chance to succeed.

**Figure 4:**
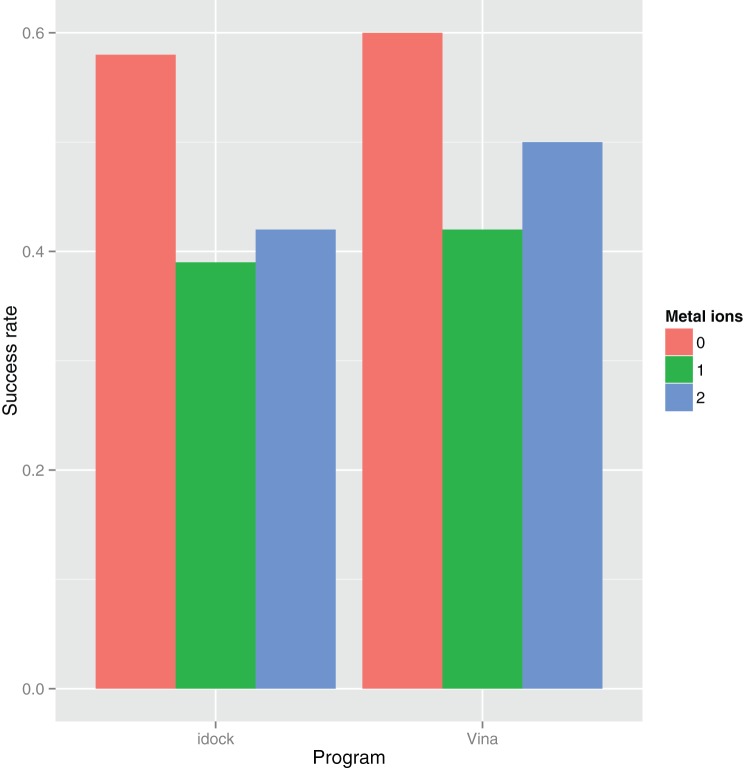
Impact of number of metal ions in the binding site on the success rates of idock and AutoDock Vina benchmarked on PDBbind v2012 core set (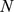 = 201). Out the 201 cases, there are 158, 31 and 12 cases in which there are 0, 1 and 2 metal ions respectively in the binding site. Very often the 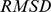 of 2.0Å is regarded as the positive control for correct bound structure prediction. For idock, the success rates are 0.58, 0.39 and 0.42 when there are 0, 1 and 2 metal ions respectively in the binding site. For AutoDock Vina, they are 0.60, 0.42 and 0.50 respectively. Docking a ligand with no metal ions in the binding site has a higher chance to succeed.

Figure 3 plots the impact of rotatable bonds of the ligand on the success rates. Both programs tend to do well when the ligand contains fewer than 10 rotatable bonds. Figure 4 plots the impact of metal ions in the binding site on the success rates. Both programs tend to do well when the binding site contains no metal ions.

**Figure 5:**
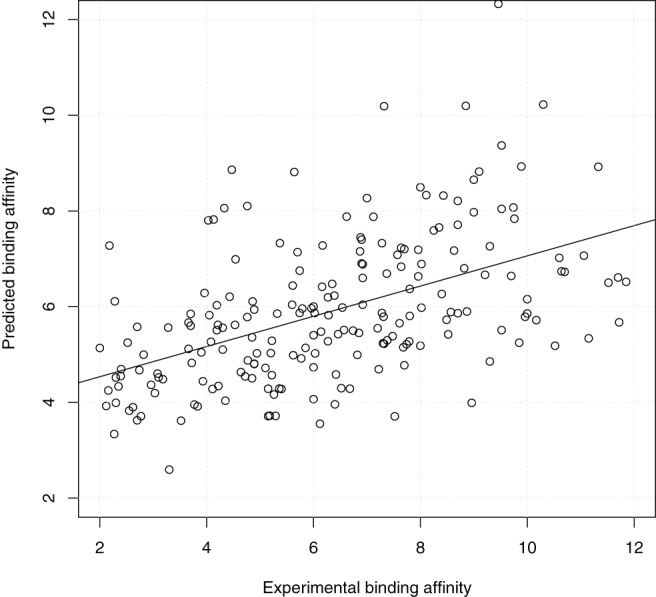
Scatter plot of the lowest idock score of the 9 docked conformations output by idock against the experimental binding affinity on PDBbind v2012 core set (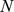 = 201) in the redocking benchmark. Values are in 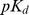 or 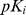 unit. 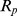 = 0.502, 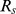 = 0.530, 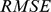  = 1.31, 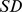  = 1.32.

**Figure 6:**
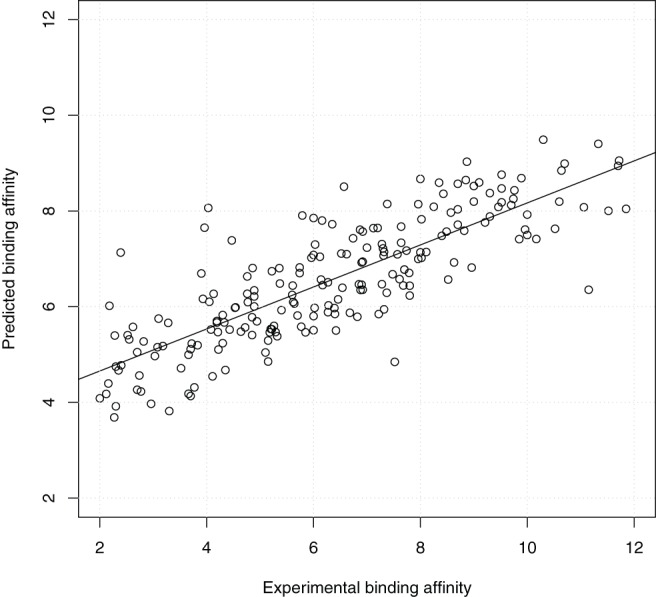
Scatter plot of the highest RF-Score of the 9 docked conformations output by idock against the experimental binding affinity on PDBbind v2012 core set (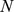 = 201) in the redocking benchmark. The RF-Score was re-trained on PDBbind v2012 refined set (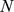 = 2,897) for prospective prediction purpose. Values are in 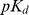 or 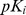 unit. 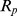 = 0.815, 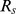 = 0.817, 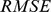  = 0.75, 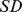  = 0.76.

**Figure 7:**
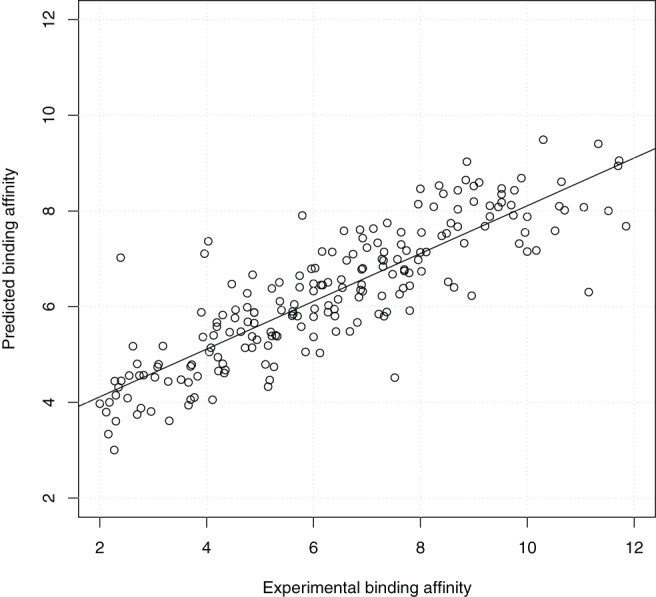
Scatter plot of the RF-Score of the first docked conformation against the experimental binding affinity on PDBbind v2012 core set (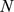 = 201) in the redocking benchmark. The RF-Score was re-trained on PDBbind v2012 refined set (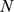 = 2,897) for prospective prediction purpose. Values are in 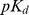 or 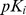 unit. 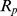 = 0.855, 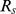 = 0.859, 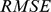  = 0.73, 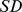  = 0.73.

From the perspective of prospective docking, Figure 5 shows the scatter plot of the highest predicted binding affinity of the 9 docked conformations output by idock against the experimental binding affinity. The weak correlation and large deviation (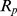 = 0.502, 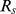 = 0.530, 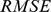  = 1.31, 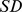  = 1.32) reflect the limitation of using idock alone as scoring function. After adopting the maximum RF-Score of the 9 docked conformations as predicted binding affinity, the correlation improves (Figure 6, 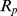 = 0.815, 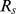 = 0.817, 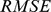  = 0.75, 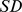  = 0.76). Moreover, since for over 50% probability the docked conformation with the highest predicted binding affinity indeed turns out to be the closest to the crystal conformation (i.e. 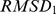  =  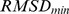), using RF-Score to re-score the conformation with 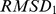 leads to even better prediction (Figure 7, 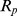 = 0.855, 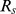 = 0.859, 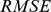  = 0.73, 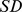  = 0.73).

### Execution Time Benchmark

Table 3 compares the CPU time and elapsed time of AutoDock Vina and idock. The execution time varied a lot from protein to protein and from molecular weight set to molecular weight set. In conclusion, idock outperformed AutoDock Vina by at least 8.69 times and at most 37.51 times, making idock particularly ideal for large-scale docking, as is the case of istar.

**Table 3:** Docking execution time.

	200–300g/mol		300–400g/mol		400–500g/mol	
	CPU	Elapsed	CPU	Elapsed	CPU	Elapsed
**1HCL** human cyclin-dependent kinase 2						
Vina	12.57	3.33	22.55	5.91	51.62	13.41
idock	0.63	0.16	0.92	0.24	1.38	0.36
**1J1B** human tau protein kinase I						
Vina	9.07	2.47	14.69	3.92	32.28	8.49
idock	0.78	0.21	1.25	0.33	2.35	0.62
**1LI4** human S-adenosylhomocysteine hydrolase						
Vina	11.82	3.30	19.08	5.22	39.41	10.64
idock	0.89	0.23	1.55	0.40	3.15	0.82
**1V9U** human rhinovirus 2 coat protein VP1						
Vina	9.80	2.95	15.55	4.62	29.75	8.49
idock	0.97	0.25	1.64	0.42	3.42	0.89
**2IQH** influenza A virus nucleoprotein NP						
Vina	9.51	2.66	15.03	4.08	29.64	7.83
idock	0.92	0.24	1.59	0.41	3.41	0.88
**2XSK** Escherichia coli curli protein CsgC – SeCys						
Vina	10.44	2.71	17.89	4.61	40.58	10.41
idock	0.71	0.19	1.16	0.30	2.16	0.56
**2ZD1** HIV-1 reverse transcriptase						
Vina	9.78	2.70	17.67	4.76	42.03	11.33
idock	0.97	0.25	1.52	0.39	2.60	0.69
**2ZNL** influenza virus RNA polymerase subunit PA						
Vina	9.49	2.60	15.04	4.01	29.97	7.82
idock	0.89	0.23	1.56	0.40	3.41	0.87
**3BGS** human purine nucleoside phosphorylase						
Vina	9.59	2.57	16.50	4.37	38.42	10.14
idock	0.95	0.25	1.55	0.40	2.81	0.74
**3H0W** human S-adenosylmethionine decarboxylase						
Vina	9.85	2.64	17.67	4.70	41.69	11.04
idock	0.88	0.23	1.35	0.35	2.20	0.58
**3IAR** human adenosine deaminase						
Vina	11.25	3.03	20.21	5.39	46.93	12.53
idock	0.80	0.21	1.21	0.32	2.01	0.53
**3KFN** HIV protease						
Vina	10.53	2.80	18.37	4.83	42.43	11.03
idock	0.77	0.20	1.20	0.32	2.09	0.55
**Average across the above 12 receptors**						
Vina	10.31	2.81	17.52	4.70	38.73	10.26
idock	0.85	0.22	1.38	0.36	2.58	0.67

CPU time and elapsed time in hours of docking 3,000 clean ligands of 3 molecular weight sets against 12 diverse receptors by AutoDock Vina and idock. idock outperforms AutoDock Vina by at least 8.69 times and at most 37.51 times.

## Discussion

Docking is the computational method that investigates how a ligand binds to a protein, and predicts their binding affinity. Hence docking is useful in elaborating inter-molecular interactions and enhancing the potency and selectivity of binding in subsequent phases of the drug discovery process.

In this study, we report a web platform called istar to automate large-scale protein-ligand docking using our popular docking engine idock. Since the initial release of idock, we have been further improving its docking speed and robustness. Compared to AutoDock Vina, our idock features a new numerical model in approximation of the scoring function, replacing slow linear interpolation by fast table lookup. It encapsulates a unique feature that can safely deactivate certain torsions to reduce the dimension of variables. It also implements an efficient thread pool to parallelize multiple components of the program and maintain a high CPU utilization. Results show that idock managed to predict a conformation sufficiently close to that of the co-crystallized ligand as the first conformation in over half of the test cases across a number of diverse datasets, and it outperformed AutoDock Vina by an order of magnitude in terms of docking efficiency at no significant cost of accuracy. It is worthwhile to highlight that in order to use istar, the input protein model requires no manual preprocessing in most cases.

We examine two possible reasons that might cause idock to fail in some test cases. They are the number of rotatable bonds of the ligand (Figure 3) and the number of metal ions in the binding site (Figure 4). On one hand, a large number of rotatable bonds implies a high dimension of variables to optimize. idock has a higher chance to succeed when the ligand consists of fewer than 10 rotatable bonds. On the other hand, all kinds of metal ions are simply treated as hydrogen bond donors in the idock score, which might not thoroughly accounts for their solvation effects and other possible interactions. idock has a higher chance to succeed when the binding site consists of no metal ions.

Although idock performs well in conformation prediction, it displays its weakness in binding affinity prediction. In contrast, RF-Score, a new scoring function that circumvents the need for problematic modelling assumptions via non-parametric machine learning, has been recently shown to obtain the best scoring performance among 16 classical scoring functions on PDBbind v2007 core set (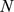 = 195) [31]. We have therefore integrated a revised version of RF-Score as an alternative re-scoring function. We have re-trained RF-Score on the entire PDBbind v2012 refined set (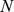 = 2,897) for prospective prediction purpose. Results show that using RF-Score to re-score the predicted conformations leads to a much better prediction with 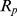 = 0.855, 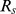 = 0.859, 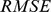  = 0.73, and 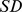  = 0.73. We have successfully demonstrated that RF-Score is a particularly effective re-scoring function for docking purposes.

To compile a more complete list of scoring functions benchmarked on the PDBbind v2007 core set (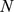 = 195) into Table 1, we have extracted the performance results for 19 scoring functions from [31], [45], [46], [48], and reported the results for AutoDock Vina and idock on the same test set in this study. This procedure has a number of advantages. Evaluating all the scoring functions on the same test set under the same conditions guarantees a fair and objective comparison. Using a common existing benchmark can also ensure the optimal application of such functions by their authors and avoid the danger of constructing an in-house benchmark on which unrealistically high performance might be produced. Moreover, future scoring functions can be unambiguously incorporated into this comparative assessment. Notably, the top four scoring functions, namely RF-Score [31], ID-Score [45], SVR-Score [46] and X-Score [47], are the only scoring functions whose training set do not overlap with the PDBbind v2007 core set. The prediction power of RF-Score is already superior to many scoring functions in commercial docking software. In terms of implementation complexity, a descriptor in RF-Score is just the occurrence count of a particular protein-ligand atom type pair interacting within a certain distance range, while a descriptor in ID-Score can be as mathematically demanding as, for instance, calculating the cosine value of the bond angle between a hydrogen bond donor and a hydrogen bond acceptor. This again demonstrates the adaptiveness of RF-Score to various applications.

One may argue that although the scoring functions are evaluated on the same test set, their training sets are not identical. Besides, the PDBbind v2007 core set consists of merely 195 complexes, which might not cover sufficient protein-ligand diversity from the perspective nowadays. To address this issue, we re-trained RF-Score on the PDBbind v2007 refined set (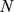 = 1,300), on which AutoDock Vina and idock were also trained, and we expanded the test set to the much larger PDBbind v2012 refined set (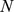 = 2,897). The results of Figure 1 show that all the performance gain (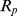 = 0.765, 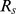 = 0.755, 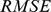  = 1.26, 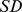  = 1.26 for RF-Score versus 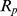 = 0.451, 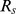 = 0.453, 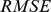  = 1.75, 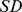  = 1.75 for idock) is guaranteed to come from the scoring function characteristics, ruling out any influence of using different training sets on performance.

To design the istar platform in a user-friendly way, we have utilized state-of-the-art web and database technologies. On istar, there are over 17 million ready-to-dock ligands collected from ZINC [20], [21]. These ligands come with supplier information for easy purchase, and they can be filtered by nine molecular properties in a fine-grained manner. The number of ligands to dock can also be previewed in real time. The jobs are transparently split into slices for parallel docking across multiple workstations, and the job progress can be monitored in real time in a browser so that users can have a rough estimation of how long the job will take and when the job will complete. Additionally, our web server supports REST API so that developers can easily submit multiple jobs in batch. Automation is the major reason of submitting jobs to istar instead of running idock locally on one's computer. With istar at hand, users need not to write special scripts to fetch ligands from some sources, to implement parallelism, or to invoke RF-Score externally by themselves. Users can therefore concentrate on the docking results and subsequent analysis rather than the docking process itself.

We compare our istar to DOCK Blaster [18], an expert system created to investigate the feasibility of full automation of large-scale protein-ligand docking. It uses DOCK [19] as the docking engine and ZINC [20], [21] as the ligand repository. Although DOCK is open source, DOCK Blaster itself is not open source. istar is indeed much easier to use than DOCK Blaster. Given the structure of a target protein, both istar and DOCK Blaster can dock and score a large set of ligands against the target protein and provide a ranked list which users may review and prioritize for purchase and wet-lab testing. From the perspective of binding site indication, istar automatically detects a site from the co-crystallized ligand, while DOCK Blaster makes use of PocketPickker (CLIPPERS) [22]. From the perspective of ligand selection, istar features ligand filtering by nine desired molecular properties in a fine-grained fashion, while DOCK Blaster predefines several subsets either by property, by vendor, or by user. From the perspective of documentation and user manual, the istar website presents a graphical tutorial on how to submit a new job, while DOCK Blaster deploys a wiki with very rich contents covering all the procedures of DOCK Blaster. As extra features, DOCK Blaster allows the input of known active and inactive binders as heuristic information for docking. In summary, although istar and DOCK Blaster share the identical motivation of automating large-scale protein-ligand docking, their internal implementations and methodologies differ greatly. Users are encouraged to utilize both istar and DOCK Blaster to reach a consensus of promising candidate ligands for purchase.

Due to limited budget, we cannot offer as much hardware resource as DOCK Blaster (i.e. 700 CPU cores plus 20TB RAID-6 storage). However, we emphasize full reproducibility and we have released istar under a permissive open source license so that anyone who possesses sufficient hardware resource is welcome to deploy a copy of istar to his/her own infrastructure with no charge.

## Availability

We emphasize full reproducibility. Both idock and istar are free and open source under Apache License 2.0. For idock, its C++ source code, precompiled executables for 32-bit and 64-bit Linux, Windows, Mac OS X, FreeBSD and Solaris, 13 docking examples, and a doxygen file for generating API documentations are available at https://github.com/HongjianLi/idock. For istar, its C++ and JavaScript source code and REST API documentation are available at https://github.com/HongjianLi/istar. Our istar website is running at http://istar.cse.cuhk.edu.hk/idock. It has been tested successfully in Chrome 30, Firefox 25, Safari 6.1 and Opera 17. Support for IE 11 is experimental.

## Supporting Information

Figure S1 **The overall architecture of istar.**
(PNG)

Figure S2 **istar supports filtering ligands with molecular properties in a fine-grained manner and previewing the number** **of ligands to dock in real time.**
(PNG)

Figure S3 **istar supports monitoring job progress in real time.**
(PNG)

Figure S4 **istar writes verbose output to file in PDBQT format.**
(PNG)

Figure S5 **Redocking result of PDB ID 1B8N.**
(PNG)

Figure S6 **Redocking result of PDB ID 4TMN.**
(PNG)

Figure S7 **Redocking result of PDB ID 1PKX.**
(PNG)

Figure S8 **Redocking result of PDB ID 3HV8.**
(PNG)
