# Supplementary figures and images for: istar: A Web Platform for Large-Scale Protein-Ligand Docking
Source: PLoS One. 2014 Jan 24;9(1):e85678. doi: 10.1371/journal.pone.0085678 (PMC3901662; doi:10.1371/journal.pone.0085678)

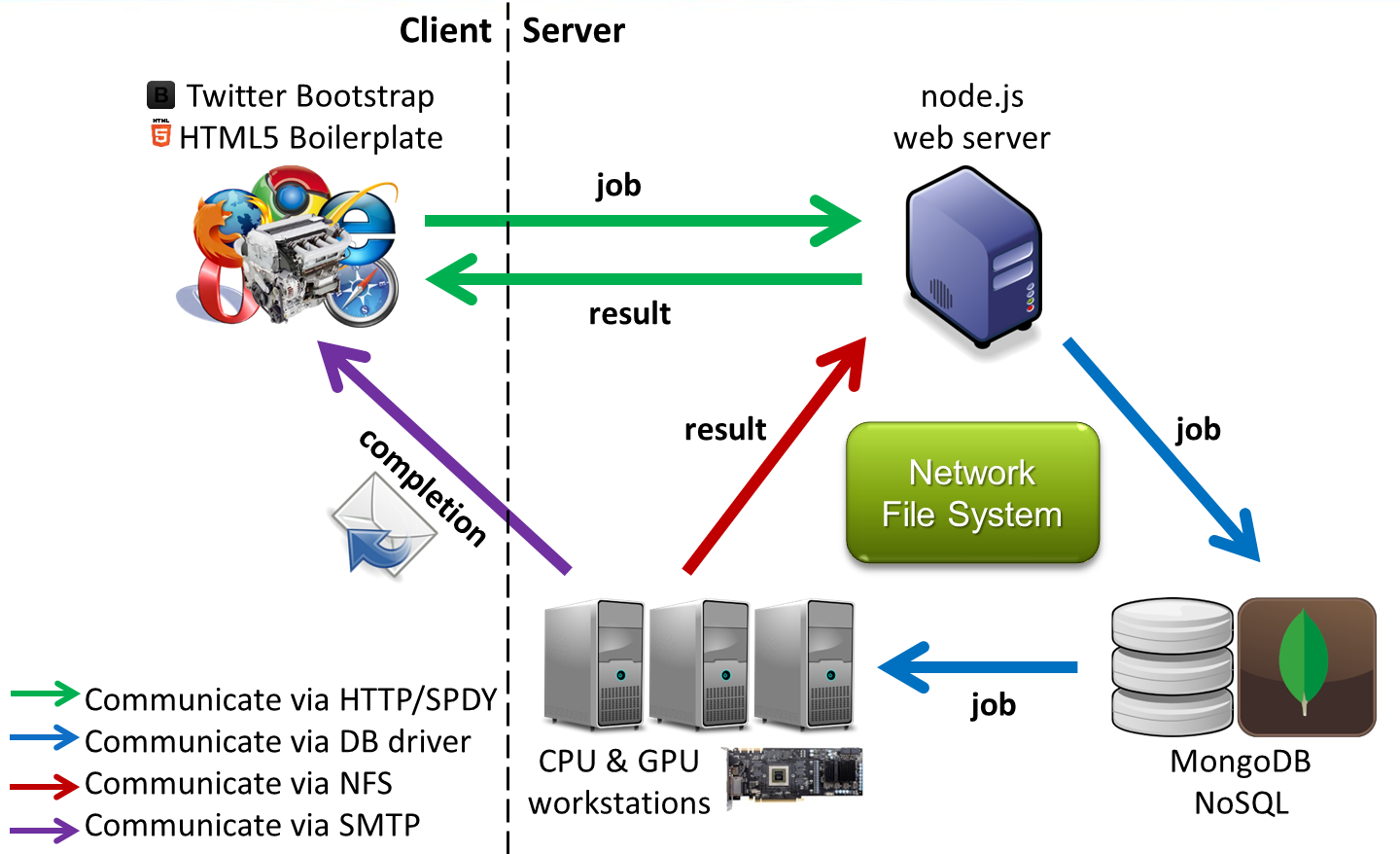

Supplement: Figure S1 — The overall architecture of istar. (PNG) [file pone.0085678.s001.png]

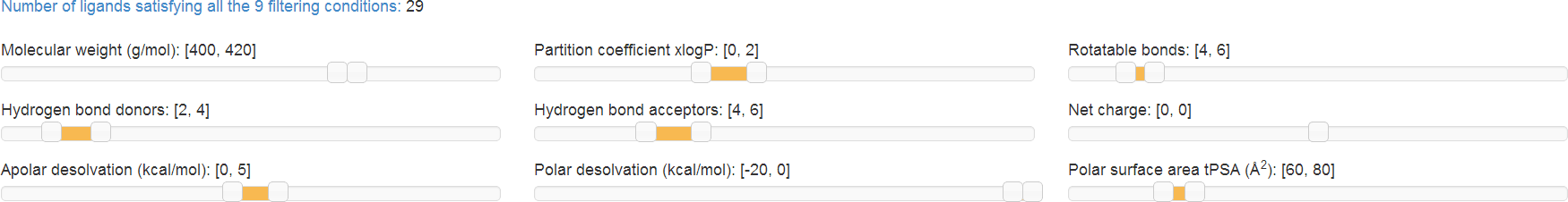

Supplement: Figure S2 — istar supports filtering ligands with molecular properties in a fine-grained manner and previewing the number of ligands to dock in real time. (PNG) [file pone.0085678.s002.png]

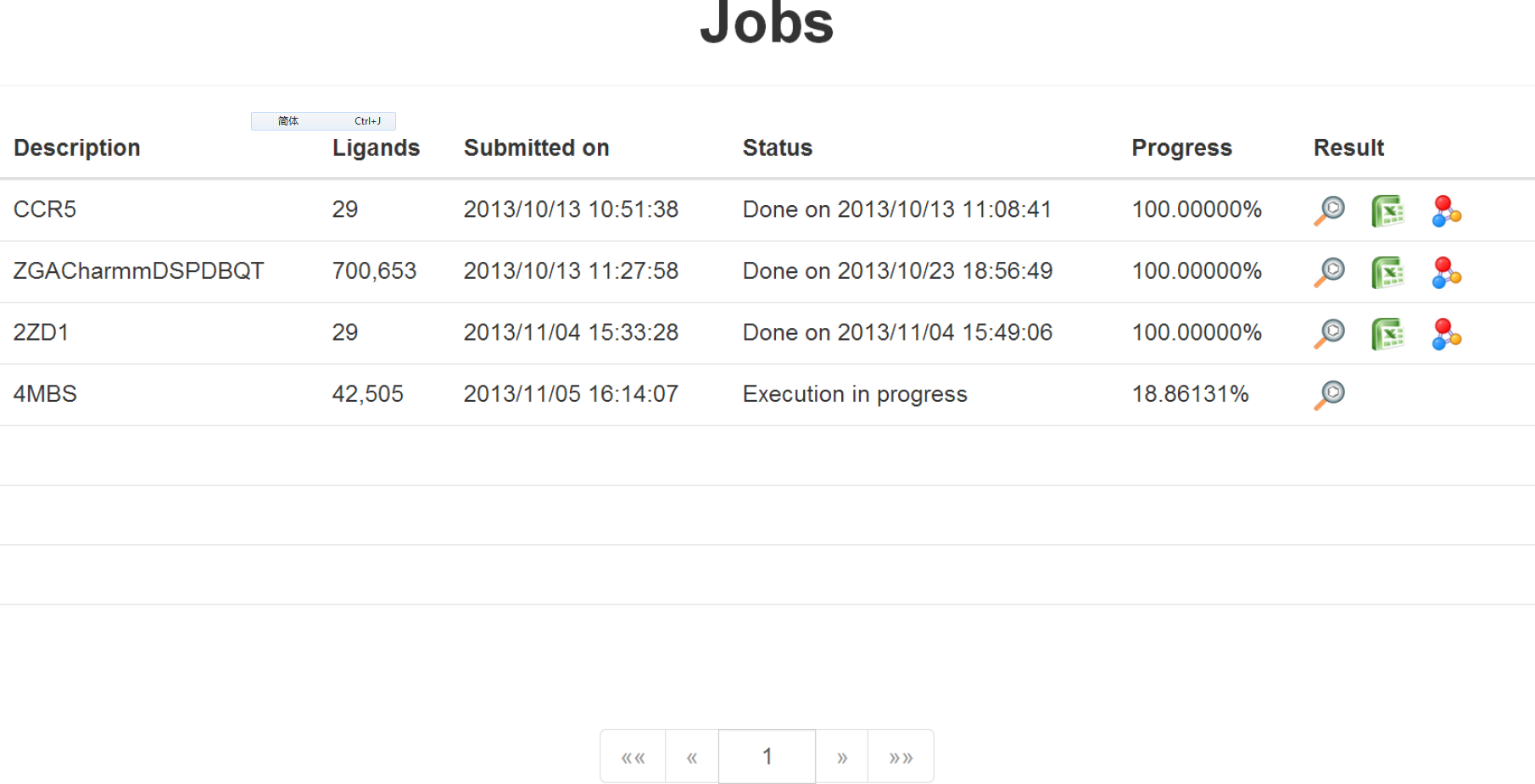

Supplement: Figure S3 — istar supports monitoring job progress in real time. (PNG) [file pone.0085678.s003.png]

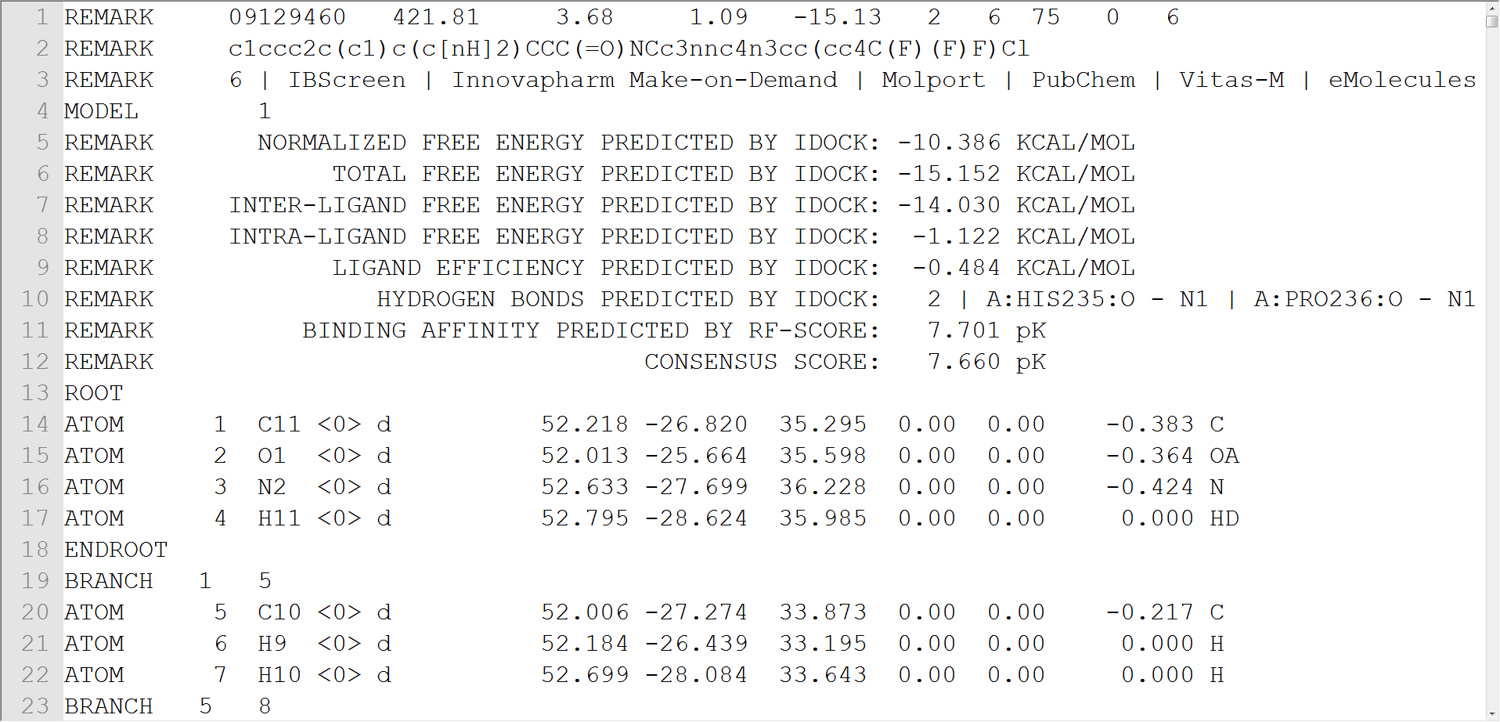

Supplement: Figure S4 — istar writes verbose output to file in PDBQT format. (PNG) [file pone.0085678.s004.png]

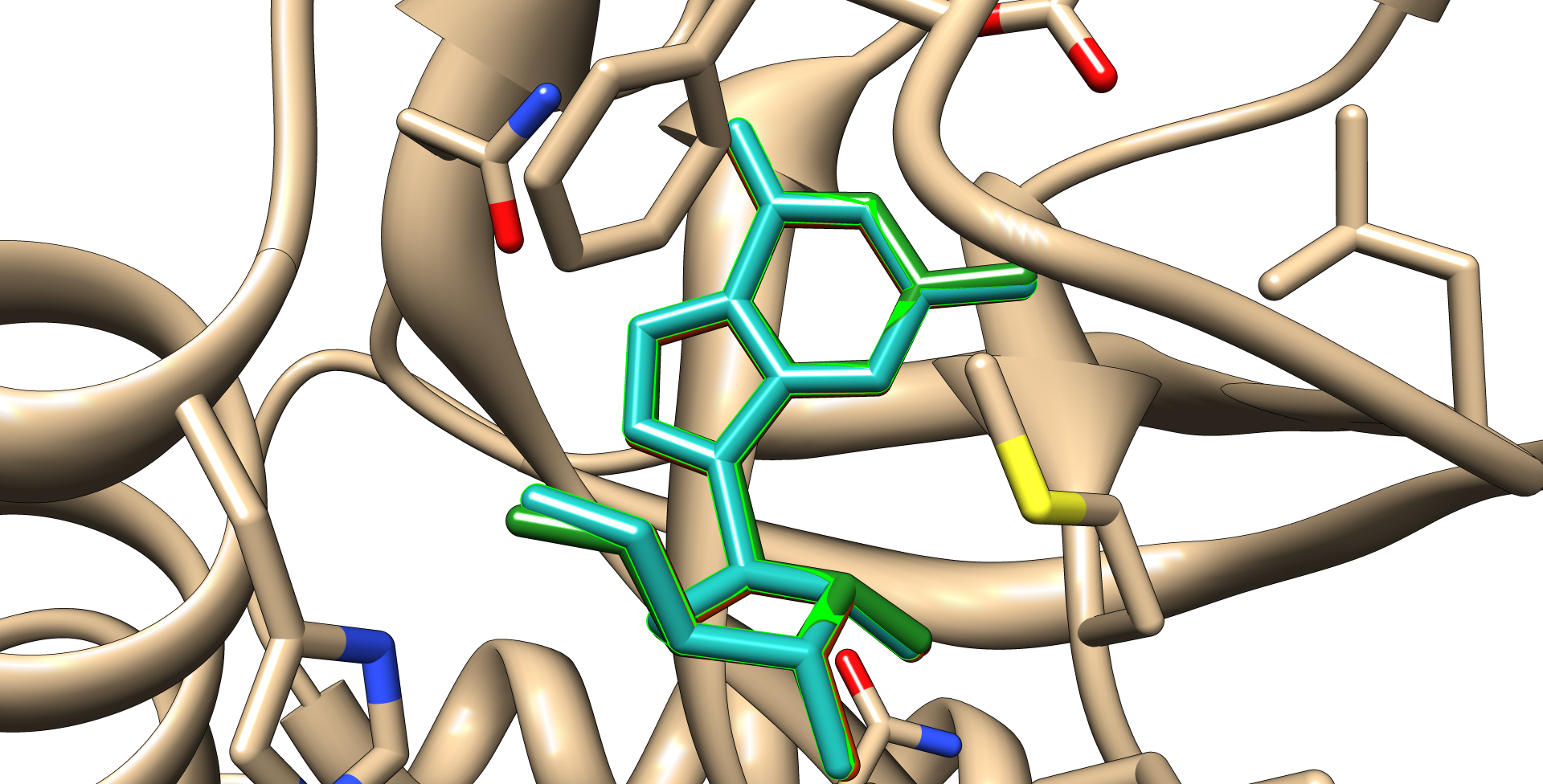

Supplement: Figure S5 — Redocking result of PDB ID 1B8N. (PNG) [file pone.0085678.s005.png]

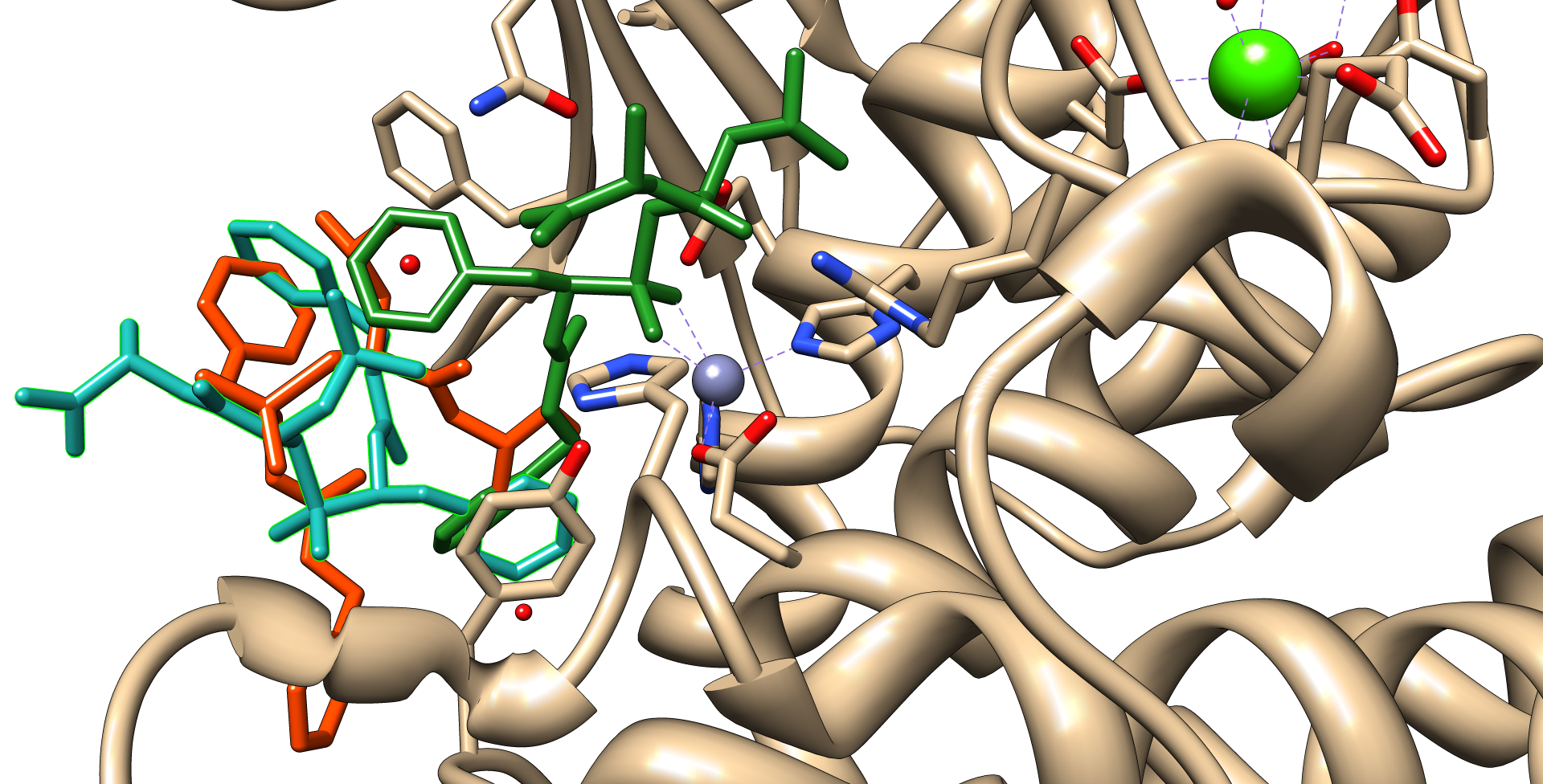

Supplement: Figure S6 — Redocking result of PDB ID 4TMN. (PNG) [file pone.0085678.s006.png]

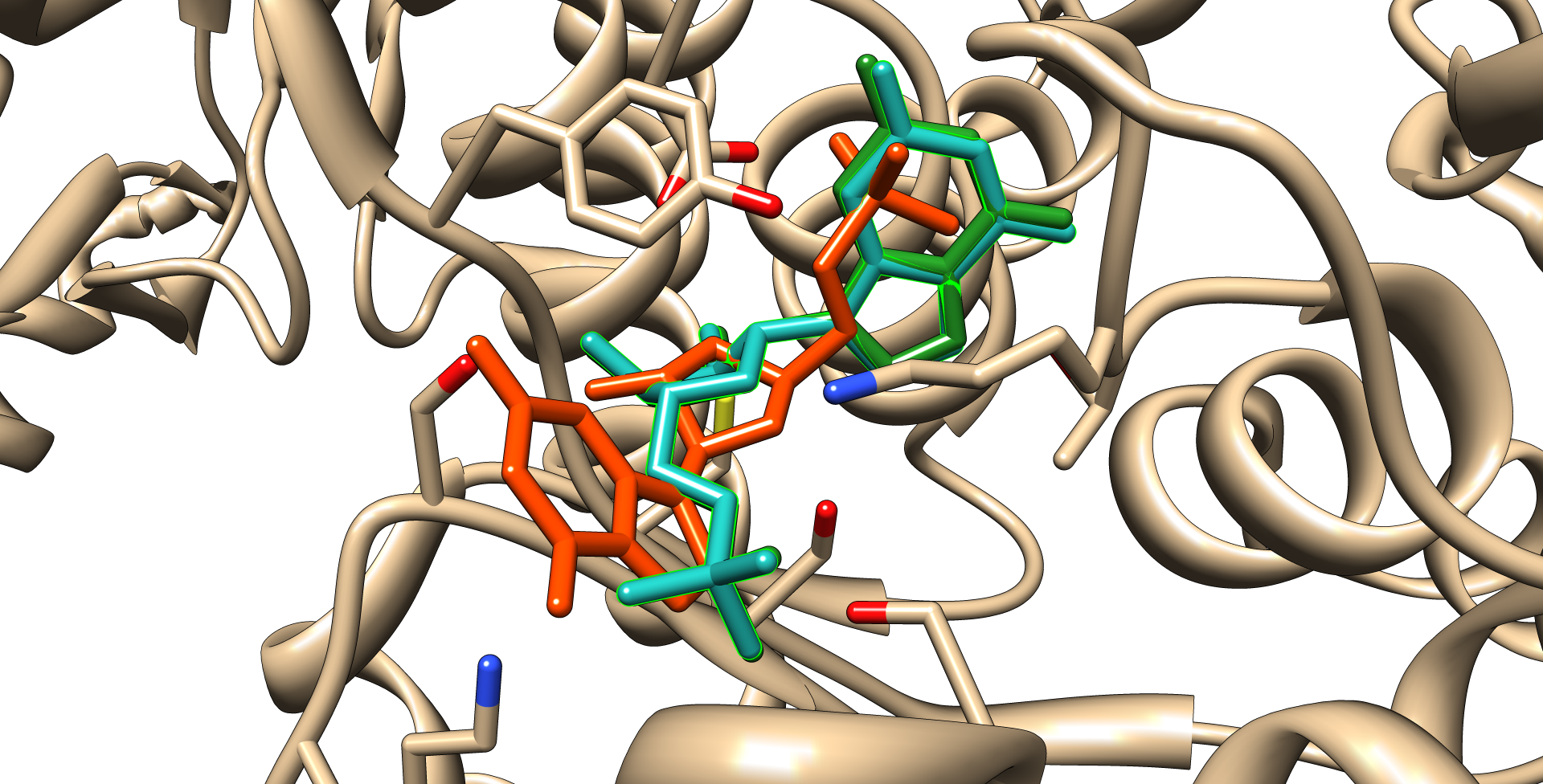

Supplement: Figure S7 — Redocking result of PDB ID 1PKX. (PNG) [file pone.0085678.s007.png]

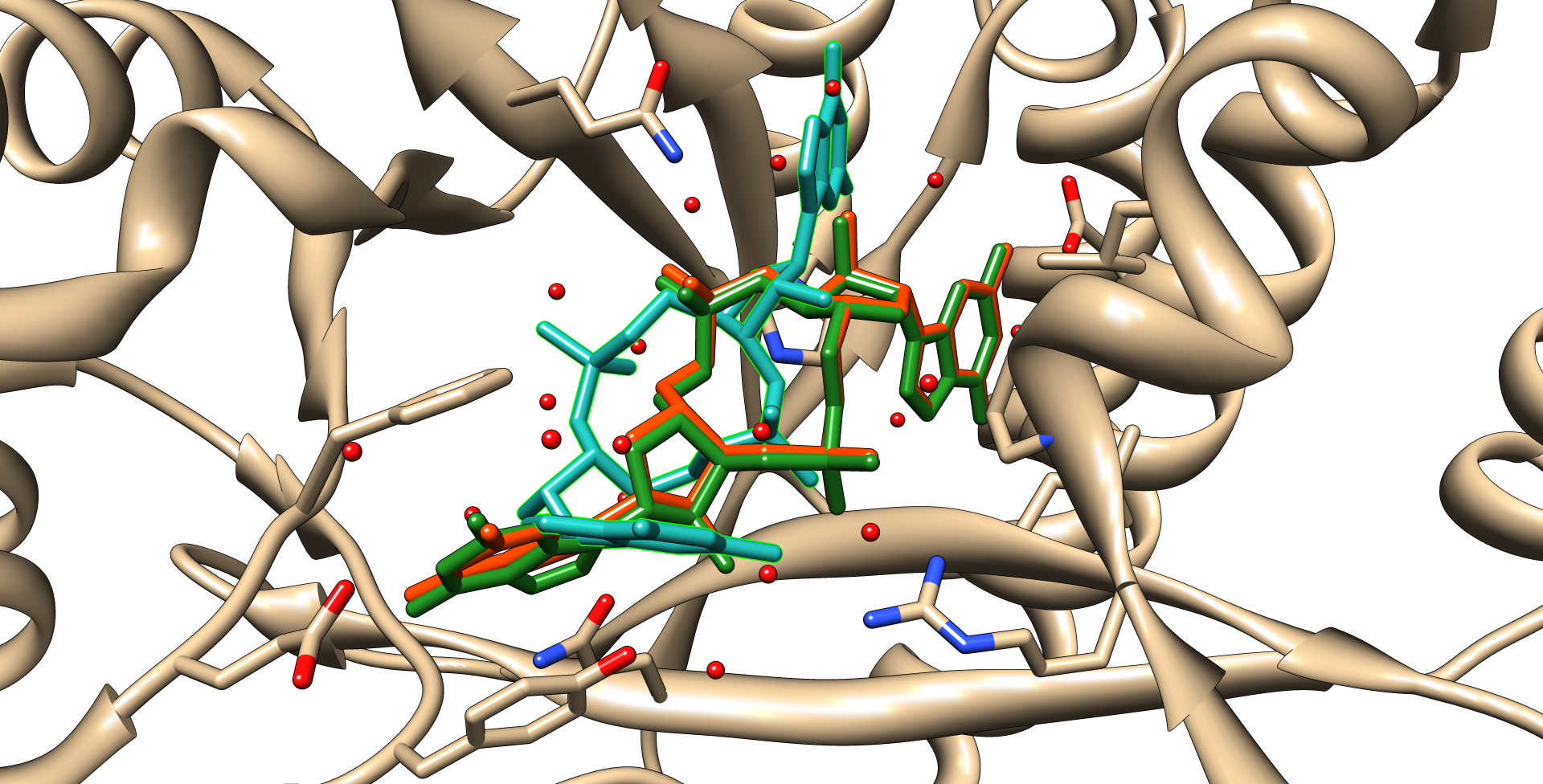

Supplement: Figure S8 — Redocking result of PDB ID 3HV8. (PNG) [file pone.0085678.s008.png]
